# Der Jenaer-Stand-Stabilitäts-Score (JESS-Score): Entwicklung, Normwertgenerierung und klinische Anwendbarkeit eines Scores zur Evaluation der Standstabilität

**DOI:** 10.1007/s00393-020-00765-8

**Published:** 2020-03-10

**Authors:** N. Best, M. Nisser, D. Loudovici-Krug

**Affiliations:** 1 Institut für Physiotherapie, Universitätsklinikum Jena, Am Klinikum 1, 07747 Jena, Deutschland; 2 Institut für Physiotherapie, Posture and Motion Group, Universitätsklinikum Jena, Jena, Deutschland; 3 Forschungsberatungsstelle der Ärztevereinigung für Manuelle Medizin (ÄMM), Am Klinikum 1, 07747 Jena, Deutschland

**Keywords:** Funktionelles Gleichgewicht, Standstabilität, Posturale Kontrolle, Assessment, Sensomotorik, Chronischer Schmerz, Functional Balance, Standing stability, Postural control, Assessment, Sensorimotor system, Chronic pain

## Abstract

**Hintergrund:**

Der Stand bzw. das Stehen kann neben den von Janda beschriebenen Bewegungsstereotypen ebenfalls als motorischer Prozess begriffen werden. Atypische Belastungen während des Stehens führen zur Überbeanspruchung myofaszialer Strukturen und zu Schmerz. Die Suche nach einer dezidierten Untersuchungsmöglichkeit mit der Aussicht auf individuelle Therapieempfehlungen, war Anlass für die Erarbeitung dieses Scores.

**Methodik:**

Es wurden 80 gesunde Probanden mittels etablierter sowie anteilig neu beschriebener Testverfahren auf ihre Standstabilität hin untersucht. Die gleichgewichteten Ergebnisse wurden zu einem Score zusammengefasst und dessen Normwerte bestimmt.

**Ergebnisse:**

Für die Altersklasse der 18- bis 44-Jährigen ist die Norm das Erfüllen von 10 der insgesamt 13 Einzelaufgaben. Für die 45- bis 59-Jährigen sind nach aktuellen Messungen 8 von 13 erreichten Punkten die Norm. In der Altersgruppe ab dem 60. Lebensjahr können bisher keine belastbaren Aussagen getroffen werden.

**Diskussion:**

Belastbare Daten liefert die Altersgruppe bis 44 Jahre. Die Altersgruppe darüber zeigt zumindest einen deutlichen Trend. Die existierenden Tests bzw. Scores setzten sich verstärkt mit dem Sturzrisiko und der Geschicklichkeit bei Bewegungen und komplexen Aufgaben auseinander. Der Stand als motorischer Stereotyp wurde bisher noch nicht beschrieben. Nach einer Untersuchung mittels Jenaer-Stand-Stabilitäts-Score (JESS-Score) ist es möglich, Aussagen zu individuellen Therapieschwerpunkten zu treffen.

**Schlussfolgerungen:**

Der JESS-Score stellt einen praktikablen Test zur Verifizierung des Standstereotyps dar. Die Erweiterung der Normgruppe durch Einschluss weiterer Studienteilnehmer wird über eine Verstetigung oder Modifikation der aktuellen Ergebnisse entscheiden. Die Testung weiterer Kohorten wird zeigen, inwieweit diese Items sensitiv für Veränderungen durch Trainingsmethoden sind und ob mit dem Score auch klinische Änderungen kongruent abgebildet werden können.

## Abstract (en)

**Background:**

Standing can be understood as a motor process in addition to the stereotypes of movement described by Janda. Atypical stress during standing leads to overstraining of myofascial structures and to pain. The search for a specific examination possibility with the prospect of individual therapy recommendations was the reason for the development of this score.

**Methodology:**

In this study 80 healthy volunteers were examined for their stance stability by means of established as well as proportionally newly described test procedures. The equally weighted results were combined into a score and its standard values were determined.

**Results:**

For the age group 18–44 years old the norm is the completion of 10 out of the total of 13 individual tasks. For the age group 45–59 years old, according to current measurements 8 out of 13 achieved points are the norm. In the age group from the age of 60 years onwards, no reliable statements can so far be made.

**Discussion:**

The age group up to 44 years old provided reliable data. The age group above that shows at least a clear trend. The existing tests and scores are increasingly concerned with the risk of falling and the dexterity in movements and complex tasks. The status as a motor stereotype has not yet been described. After an examination using the Jena standing stability (JESS) score it is possible to make statements about individual therapy priorities.

**Conclusion:**

The JESS score is a practicable test to verify the standing stereotype. The extension of the norm group by including further study participants will decide on a stabilization or modification of the current results. The testing of further cohorts will show to what extent these items are sensitive to changes caused by training methods and whether the score can also be used to congruently map clinical changes.

Schmerzen im Bewegungssystem entstehen häufig durch Über- oder Fehlbelastung. Ursächlich können neben den Störungen der Stereotype allgemeiner Bewegungsabläufe auch die des Stehens sein. Der Stand wurde aber bislang kaum als motorische Aufgabe betrachtet. Mit dem hier vorgestellten JESS-Score ist es möglich, die notwendigen motorischen Fähigkeiten für das Stehen differenzierter zu untersuchen als bisher. Durch die neu gewonnenen Normwerte können nun konkrete Behandlungsoptionen und damit gezielte Therapievarianten eingeleitet werden.

## Hintergrund und Fragestellung

Der aufrechte Stand des Menschen ist ein dynamischer Prozess [62]. Das Bewegungssystem sichert diese aufrechte Position um einen Mittelpunkt (Lot) entgegen der Schwerkraft. Dieser Punkt wird „Center of Pressure“ (COP) genannt. Um die Funktion des Stehens zu gewährleisten, sind stereotype Bewegungsmuster notwendig [5], die permanent die Haltung korrigieren und das Umfallen verhindern. Wie bei allen Bewegungsstereotypen ist es auch beim Stehen möglich, Muskulatur in einer unökonomischen bzw. unphysiologischen Art und Weise anzusteuern. Die Folge daraus können Überlastung und Übersäuerung der Muskulatur bis hin zu Schmerzen sein [29]. Störungen der Haltung und Bewegung mit resultierendem Schmerz sind ein häufig anzutreffendes Leiden in der deutschen Bevölkerung [9, 11, 17]. Eine Vielzahl von Patienten mit derartigen muskuloskeletalen Beschwerden stellt sich rheumatologischen Kollegen vor. Dies betrifft sowohl internistisch als auch orthopädisch ausgebildete Fachkollegen. Des Weiteren werden diese Patienten in hoher Zahl auch beim Hausarzt vorstellig [50]. Chronische Schmerzsyndrome sind gesundheitsökonomisch äußerst relevant [48]. Eine möglichst zielgerichtete Therapie dient hierbei der Kosteneinsparung [12]. Der hier vorgestellte Jenaer-Stand-Stabilitäts-Score (JESS-Score) soll helfen, Patienten mit Stereotypstörungen während des Stehens und die daraus resultierenden Beschwerden am Bewegungssystem zielgerichteter zu diagnostizieren und zu behandeln. Das Ziel der Score-Entwicklung war die Erarbeitung eines klinisch anwendbaren Tools, das in der täglichen Praxis einsetzbar ist.

Die Grundlage bildet die Tatsache, dass die Stabilität des Stehens, ebenso des Gehens, durch mehrere motorische Fähigkeiten generiert wird [31, 58]. Beschrieben wurden diese grundlegend durch Meinel und Schnabel [35]. Für den Patienten ist allerdings das klinische Gesamtergebnis wichtig, die Stabilität bzw. Unsicherheit und ggf. aufgetretene Sturzereignisse. Mittlerweile werden Muskel-Gelenk-Systeme nicht mehr isoliert betrachtet oder Gelenke alleinig als mechanische Komponente angesehen. Unter Würdigung neuer Erkenntnisse zur Funktion von Faszien ist es nunmehr unbestritten, dass myofasziale Ketten an Bewegungsausführung, biomechanischen Vorgängen und Ökonomisierung von Bewegungsabläufen beteiligt sind [39, 42]. Bislang befassen sich andere Messsysteme eher mit dem Sturzrisiko beim Gehen, oder die Auswertung ist nur undifferenziert möglich [2–4, 16, 43, 47, 51].

## Studiendesign und Untersuchungsmethoden

Thomas Myers beschreibt in seinem Fachbuch verschiedene myofasziale Ketten, die bei Bewegungsausführung und aufrechter Körperhaltung wichtig sind und auch im JESS-Score eine große Rolle spielen. Es wurden die anteriore und posteriore Kette sowohl hinsichtlich der Kraftausdauer als auch der Bewegungskoordination getestet. Zweifelsfrei sind diese Ketten nicht isoliert ansprechbar, sodass davon ausgegangen werden kann, dass alle spiralig und seitlich verlaufenden myofaszialen Bahnen in diese Bewegungsprüfung integriert sind und die Ergebnisse beeinflussen [39].

Aufgrund dieser oben genannten Aspekte entschieden sich die Autoren zur Zusammenfassung der im Folgenden aufgeführten Testverfahren zu einem Gesamtscore. Die enthaltenen Tests können nicht nur einer Entität zur Feststellung des Gleichgewichts allein zugeordnet werden, sondern bedienen mit hoher Wahrscheinlichkeit stets mehrere Bewegungsqualitäten bzw. -funktionen. Deswegen ist das Eingruppieren unter einer jeweils alleinigen Bewegungsqualität nicht zielführend. Folgend der Versuch einer Einteilung (Tab. 1).

**Table 1:** 

	Ausdauertests nach McGill [16]/Ito [11]	ZST [8]	Provokationsposturographie [27, 28]	Bregma-Test	Bewegungsstereotype nach Janda [12, 22]	EBS [24]	SF-12 [5, 14, 18]
Kraftausdauer (anterior und posterior)	x						
Differenzierungsfähigkeit		x					
Reaktionsfähigkeit			x	x			
Sensomotorik/Bewegungsstereotypie (Hüftflexion, Rumpfaufrichtung)				x	x		
Relevanz der visuellen Kontrolle		x	x			x	
Allgemeiner Gesundheitszustand, physisch und psychisch							x

*ZST* Zielschritttest, *EBS* Einbeinstandttest

### Provokationsposturographie

Durch eine geeignete Vorrichtung wird die Posturomed-Plattform in eine Richtung ausgelenkt und arretiert. Dieser Mechanismus führt anschließend zu einer Rückstellreaktion, die je nach Ausrichtung des darauf stehenden Probanden zu einer sagittalen oder frontalen Bewegung der Plattform führt. Darauf muss der Proband reagieren und die Rückstellkräfte so dämpfen, dass der aufrechte Stand für mindestens 10 s erhalten bleibt. Der Test wird beidbeinig bzw. auf einem Bein stehend durchgeführt. Weiterhin sollen die Augen im Wechsel geöffnet oder geschlossen werden. Alle Varianten werden kombiniert untersucht [60, 61].

### Zielschritttest (ZST)

Der Proband steht ohne Schuhe an einer Ausgangslinie: beim ZST nach vorn mit den Zehen und beim ZST zur Seite mit der lateralen Fußkante. Nun wird versucht mit dem Zeh bzw. der lateralen Fußkante des zu bewegenden Beines (Basis D5) einen Zielstrich zu treffen, der 25 % der Körperhöhe des Probanden von der Ausgangslinie entfernt ist. Nach 2 Versuchen mit geöffneten Augen wird der Versuch nun mit geschlossenen Augen ausgeführt und der Abstand zum Zielstrich bestimmt. Normwertig für alle Bewegungsrichtungen ist ein Bereich von ±50 mm entfernt vom Zielstrich [13, 55].

### Einbeinstandtest (EBS)

Der EBS wird mit beiden Beinen einzeln ausgeführt, sowohl mit geöffneten als auch mit geschlossenen Augen. Dabei wird die Länge des stabilen Stehens ohne Ausweichbewegungen bestimmt [53].

### Bregma-Test

Die üblicherweise eingenommene Position beim Stehen wird als Ausgangsstellung genutzt. Nach taktiler Fazilitation des Bregmas (Punkt der neonatalen zentralen Fontanelle) durch den Untersucher wird der Proband aufgefordert, sein Bregma Richtung Decke zu bewegen. Dabei wird die qualitative Bewegungsausführung bewertet. Die korrekte Bregmavertikalisierung wird, je nachdem ob mit oder ohne Parakinesen, mit Graden von 1,0–1,3 bewertet, unphysiologische Bregmabewegungen entsprechend mit Graden von 2,0–2,3 [5, 6].

### Bewegungsstereotyp der Hyperextension im Hüftgelenk

Dieses Bewegungsstereotyp wurde zuerst von Vladimir Janda beschrieben und die Auswertung mittlerweile modifiziert. In Bauchlage sollen die Beine abwechselnd gestreckt abgehoben werden, ohne dass sich der Proband mit den anderen Extremitäten abstützt. Gewertet wird die Reihenfolge der angesprochenen Muskelgruppenetagen von unten nach oben. Als physiologisch gilt die Reihenfolge Hamstring‑/Glutaeusgruppe, Erektoren der lumbalen Wirbelsäule und schließlich die thorakale Muskulatur [24, 49].

### Bewegungsstereotyp der Rumpfanteflexion

Auch dieses Bewegungsstereotyp wurde zuerst von Janda beschrieben. Die Interpretation ist seit Beschreibung unverändert. Das Anheben des Kopfes aus Rückenlage soll mit deutlicher Flexion der Halswirbelsäule erfolgen. Anschließend wird der Rumpf so gebeugt, dass die unteren Skapulawinkel mindestens 5 cm von der Unterlage abgehoben sind ohne Fersenabhebung. Dies gilt als Zeichen der Aktivierung des Psoasmuskels und somit als unphysiologisch [24, 49].

### Testung der anterioren Kette auf Kraftausdauerfähigkeit nach McGill

In einer rückwärts geneigten Sitzhaltung (30°) mit aufgerichtetem Becken sowie mit rechtwinkliger Knie- und Hüftgelenkstellung wird die Ausdauerfähigkeit der dazu benötigten Muskelgruppen geprüft. Durch McGill et al. sind Normwerte beschrieben, wobei nach 5-minütiger Testung abgebrochen wird [34].

### Testung der posterioren Kette auf Kraftausdauerfähigkeit nach Ito

Durch geeignete Unterlagerung des Beckens in Bauchlage wird es ermöglicht, den Rumpf leicht von der Unterlage abzuheben, ohne eine Verankerung der unteren Extremitäten in Anspruch zu nehmen. Eine durch die Autoren beschriebene Normzeit liegt als Testgröße vor. Analog zum Test nach McGill wird beim Erreichen einer Haltezeitdauer von 5 min der Test beendet [22].

### SF-12

Der SF-12 stellt die evaluierte Kurzform des SF-36 dar. Der SF-36 ist das weltweit meistgenutzte generische Assessment zur Erfassung des allgemeinen Gesundheitszustandes. Bei Auswertung des SF-12 kann jeweils eine Summenskala für körperliche sowie seelische Gesundheit gebildet werden [10, 30, 38].

Für die einzelnen Tests sind die Gütekriterien bzw. Normwerte den Originalpublikationen zu entnehmen. Für neue Tests wurden diese durch die Autoren ermittelt. Alle Parameter des JESS-Scores sind gleich gewichtet. Zur optimierten Auswertung dient ein Netzdiagramm. Einzelne Parameter, in welchen noch nicht die physiologische Norm erreicht wurde, werden farbig hervorgehoben. So können Defizite von Probanden bzw. Patienten auf einen Blick erkannt werden.

Untersucht wurden gesunde Erwachsene. Es wurde eine Einteilung in Alterskategorien, wie von Meinel und Schnabel vorgeschlagen, durchgeführt [35]. Als Altersgrenzen wurden immer die unteren Grenzwerte angenommen. Die zugrunde gelegte Einteilung zeigt die Tab. 2.

**Table 2:** 

Lebensabschnitt	Altersspanne	Motorische Charakteristik
Frühes Erwachsenenalter	18. bis 29. Lebensjahr	Relativer Erhalt der motorischen Lern- und Leistungsfähigkeit
Mittleres Erwachsenenalter	30. bis 44. Lebensjahr	Allmähliche motorische Leistungsminderung
Spätes Erwachsenenalter	45. bis 59. Lebensjahr	Verstärkte motorische Leistungsminderung
Alter	Ab 60. Lebensjahr	Ausgeprägte motorische Leistungsminderung

Dem vorherigen Schema folgend, wurde eine zusätzliche Stufe ab dem75. Lebensjahr eingefügt. Hintergrund ist die Annahme, dass eine regelmäßige körperliche Aktivität sich positiv auf die Lebensqualität, Gedächtnisleistung und Reaktionsfähigkeit auswirkt. Das Renteneintrittsalter liegt aktuell bei 67 Jahren. Die Erwerbstätigenquote im Alter von 55 bis 64 Jahren ist zuletzt deutlich gestiegen, und gesundherhaltende Aktivitäten werden teilweise bis ins fortgeschrittene Seniorenalter durchgeführt [7, 63, 64].

Die Baselinecharakteristik der untersuchten Studienpopulation sind der Tab. 3 zu entnehmen.

**Table 3:** 

	MW ± SD oder %
*Alter* (Jahre)	37,6 ± 11,8
*Frauen, n* (%)	51 (63,8)
*BMI* (kg/m^2^)	24,4 ± 4,1
*Spielbein*	
Rechts, *n* (%)	76 (95)
Links, *n* (%)	3 (3,75)
Beidseits, *n* (%)	1 (1,25)
*Finger-Boden-Abstand*	3 ± 6,3 cm

*MW* Mittelwert, *SD* Standardabweichung

Entsprechend der festgelegten Altersgruppierung ergibt sich folgendes Verteilungsbild in der aktuellen Untersuchungsgruppe (Tab. 4).

**Table 4:** 

18–44. Lj	56
45–59. Lj	22
60.–74. Lj	2
>75. Lj	0

Die Übersicht der Einzelitems ist in Tab. 5 dargestellt. Dabei sind neben einer Auflistung der verschiedenen Werte, auch die Normwerte benannt.

**Table 5:** 

JESS-Score, Einzeltests						Werte		Normwert		
								Vorh	Neu	Benennung
*Provokationsposturographie*										
				Augen						
Ohne Impuls Impuls von lateral Impuls von posterior	Beidseits			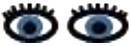		M = 3			x	3
				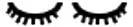		M = 3			x	3
	Einseitig			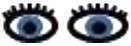		M = 6			x	6
				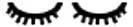		M = 1			x	1
*Zielschritttest*										
Drei Probeschritte mit geöffneten Augen Vierter Versuch mit geschlossenen Augen	Vorn			Rechts		M = 0,3			x	±5 cm
				Links		M = 0,7			x	
	Seitlich			Rechts		M = 1			x	
				Links		M = 0,5			x	
*Einbeinstandtest*										
	Augen									
Rechts Links Ohne Ausweichbewegung	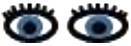		Rechts				MW = 45s	x		45 s
			Links				MW = 45s	x		
	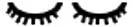		Rechts				MW = 3s	x		15 s
			Links				MW = 2s	x		
*Bewegungsstereotype nach Janda*										
Reihenfolge: 1 – untere Extremität 2 – lumbal 3 – thorakal	Hüftextension				Rechts	1‑2-3: 71 %		x		1-2‑3
					Links	1‑2-3: 71 %		x		
x‑5 cm-Abstands Scapulae, x‑ohne Rumpfsteifhaltung x‑und Hüftbeugeraktivität	Rumpfaufrichtung					x‑x-x: 80 %		x		x-x‑x
*Bregma-Test*										
1,0–1,2: physiologisch 2,0–2,2: pathologisch						M = 1,0			x	1,0
*Kraftausdauertests*										
Extension – posteriore Kette		n. Ito				MW = 226		x		m/w: 202 s/128 s
Flexion – anteriore Kette		n. McGill				MW = 193		x		m/w: 144 s/149 s
*SF-12*										
Zwei Summenskalen Körperlich/psychisch	Körperlich (KSK)					MW = 50,12		x		50 ±10
	Psychisch (PSK)					MW = 52,04		x		

*JESS-Score* Jenaer-Stand-Stabilitäts-Score, *M* Median, *MW* Mittelwert, *vorh.* vorhanden, *n* Anzahl, *KSK* körperliche Summenskala, *PSK* psychische Summenskala

### Provokationsposturographie

In der Arbeit von Wegerhoff [60] wurden erstmals Schwierigkeitsgerade bezüglich der Provokationsposturographie herausgearbeitet. Die Ergebnisse zusammenfassend und stark vereinfachend, lässt sich eine Reihung mit ansteigendem Schwierigkeitsgrad folgendermaßen definieren:

1. breitbeiniger Stand mit geöffneten Augen (unabhängig ob mit oder ohne Provokation bzw. von der Provokationsrichtung),
2. breitbeiniger Stand mit geschlossenen Augen (unabhängig ob mit oder ohne Provokation bzw. der Provokationsrichtung),
3. einbeiniger Stand mit geöffneten Augen (unabhängig ob mit oder ohne Provokation bzw. der Provokationsrichtung und unabhängig vom genutzten Bein),
4. einbeiniger Stand mit geschlossenen Augen (unabhängig ob mit oder ohne Provokation bzw. der Provokationsrichtung und unabhängig vom genutzten Bein).

Die hier generierten Normwerte stellen sich folgendermaßen dar. Für Schwierigkeitsstufe 1 bis 3 müssen immer alle Anforderungen erfolgreich absolviert werden müssen. Bezüglich Stufe 4 muss eine der darin enthaltenen Aufgaben (Linksbeinstand bzw. Rechtsbeinstand mit oder ohne Provokation unabhängig von der Richtung) geschafft werden, um die Norm zu erfüllen.

### Zielschritttest

Daten der Autoren zeigen, dass unabhängig von der Ausführung des ZST der Normwertbereich ±5 cm um den Zielstrich herum liegt [55]; 86 % aller Probanden erfüllten diese Norm (*n* = 69). Bei 11 Probanden ergab der Test pathologische Ergebnisse; 7 von ihnen erreichten den avisierten Zielbereich in einer Richtung und 4 in mehrere Richtungen nicht. In Anlehnung an die Theorien von Carl Friedrich Gauß wurde die 5. bzw. die 95. Perzentile berechnet [56]. Es zeigte sich, dass bei lediglich 3 Versuchen der physiologische Zielschrittbereich untertreten, aber 12-mal übertreten wurde. Zusammengefasst wurden die Werte in Tab. 6.

**Table 6:** 

Zielschritt nach vorn	Rechts	*n* = 4	2x −, 2x +
	Links	*n* = 1	1x +
Zielschritt zur Seite	Rechts	*n* = 4	4x +
	Links	*n* = 6	1x −, 5x +

+ übertreten, − untertreten, *ZST* Zielschritttest

Dabei gelten die mit einem Plus markierten Versuche als übertreten. Dementsprechend sind die untertretenen (also zu kurz ausgeführten) Versuche mit einem Minus dargestellt; 12 der insgesamt 15 fehlerhaften Versuche waren übertreten. Lediglich 3 Versuche wurden zu kurz ausgeführt.

### Einbeinstandtest

Die Prüfung des EBS zeigt, dass in Abhängigkeit des Alters deutliche Diskrepanzen zwischen der Ausführung mit geöffneten und geschlossenen Augen bestehen. Die vorliegende Untersuchung legt dar, dass alle Probanden die Norm für den EBS sowohl links- als auch rechtsseitig mit geöffneten Augen erreichten. Entgegen den bestehenden Normwerten für den Test mit geschlossenen Augen konnten diese Probanden im Median nur 2 s auf dem linken Bein und 3 s auf dem rechten Bein ohne Ausweichbewegungen stehen. Es zeigte sich auch, dass wenige Studienteilnehmer die Normzeit für eine Seite mit geschlossenen Augen schafften, in diesem Fall aber nicht für beide Seiten.

### Bregma-Test

Die untersuchten Probanden zeigten mehrheitlich eine physiologische Testausführung. Der Median der Kohorte lag bei 1,0. Lediglich 11 Studienteilnehmer zeigten pathologische Bewegungsmuster. Dies entspricht ca. 9 % der untersuchten Probanden.

### Bewegungsstereotyp der Hyperextension im Hüftgelenk

Der Bewegungsstereotyp Hüftextension wurde bei allen 80 Probanden geprüft. Es zeigte sich, dass sowohl links als auch rechts jeweils 23 pathologische Versuchsdurchführungen auffielen. Damit zeigten 29 % aller untersuchten Probanden eine unphysiologische Bewegungsabfolge. Es ließ sich nachweisen, dass bei 14 Untersuchten die Ausführungen beider Seiten pathologisch und bei 48 beidseits physiologisch waren. Bei den übrigen 18 Probanden konnte ein pathologischer Stereotyp auf nur einer Seite identifiziert werden.

### Bewegungsstereotyp der Rumpfanteflexion

Bei Prüfung der Rumpfanteflexion hingegen konnten 16 pathologische Bewegungsmuster gefunden werden. Dies entspricht 20 % der Untersuchten.

### Testung der anterioren Kette auf Kraftausdauerfähigkeit nach McGill

**Figure 1:**
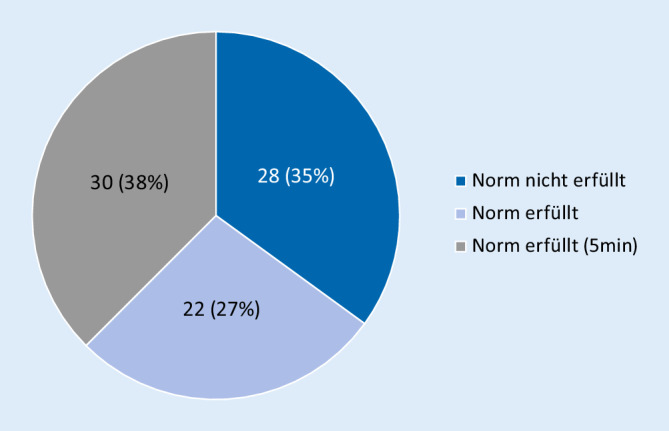

Die Testdauer bei den hier untersuchten Probanden lag im Mittel bei 193 ± 102 s. Damit zeigte sich die Mehrzahl der untersuchten Personen oberhalb der von McGill definierten Norm von 144s/149s (m/w); 28 der Untersuchten schafften die vorgegebene Norm nicht; 30 erreichten hingegen das Abbruchkriterium von 5 min (Abb. 1).

### Testung der posterioren Kette auf Kraftausdauerfähigkeit nach Ito

Im nach Ito beschriebenen Test der Kraftausdauer der posterioren Kette konnten die untersuchten Probanden im Mittel 226 s ± 86 s erreichen. Damit waren ebenfalls im Mittel die Normwerte deutlich übertroffen (202 s/128 s, m/w). Mit 22,6 % lag die Anzahl der Probanden, die unter der Norm blieben, etwas unterhalb der Werte des Tests nach McGill.

**Figure 2:**
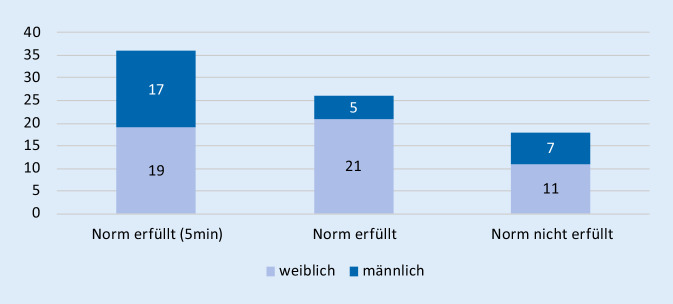

Es lagen 14 Personen aus der Kohorte sowohl bei der Testung für die anteriore als auch die posteriore Kette unter den Vorgaben: 9 Frauen und 5 Männer. Dies entspricht 17,6 % des Frauenanteils und 17,2 % der Männerkohorte (Abb. 2 und 3).

**Figure 3:**
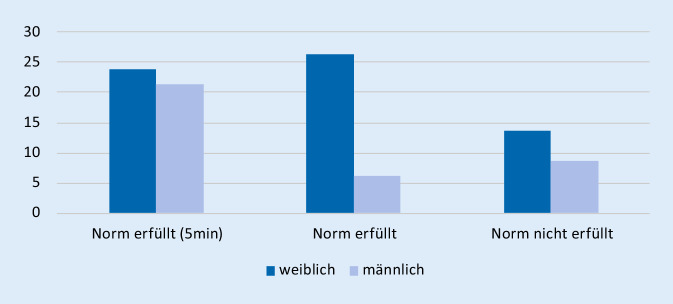

### SF-12

Der SF-12 wird in 2 Summenskalen ausgewertet: zum einen die körperliche (KSK) und zum anderen die psychische Summenskala (PSK). Die Normwerte liegen bei einem Summenscore von jeweils 50 ± 10 Punkten. In der untersuchten Kohorte zeigten 9 Probanden Werte unter 40 für die KSK und 5 Probanden für die PSK. Lediglich eine Person zeigte Werte unterhalb der Norm in beiden Summenskalen.

## Übereinstimmungen ausgewählter Tests

Der Bregma-Test und Jandas Bewegungsstereotypien (Hyperextension im Hüftgelenk, Rumpfanteflexion) prüfen ähnliche Bewegungsqualitäten. Bei pathologischen Bregma-Test-Werten zeigen sich in 66 % der Fälle ebenfalls pathologische Bewegungsausführungen der Hüftgelenkhyperextension. Bezogen auf die Rumpfanteflexion ergibt sich eine Übereinstimmung von 70 %.

Grundsätzlich lässt sich zeigen, dass Auffälligkeiten der Bewegungsstereotypien deutlich häufiger auftreten als eine pathologische Ausführung des Bregma-Tests.

Eine ähnliche Beobachtung lässt sich bei Prüfung von Übereinstimmungen zwischen Einbeinstandtest und Provokationsposturographie machen; 74 % der Untersuchten zeigten Auffälligkeiten bei der Provokationsposturographie mit geöffneten Augen, wenn der Einbeinstandtest mit geöffneten Augen pathologisch ausfiel. Für die Prüfung mit geschlossenen Augen konnten Übereinstimmungen von 62 % der Fälle ermittelt werden.

## Normwerte des JESS-Scores

Zur spezifischen Auswertung innerhalb der Altersklassen wurde initial die Klasse der 18- bis 44-jährigen ProbandInnen herangezogen. Die Norm wurde erfüllt, wenn 10 der 13 Aufgaben erfüllt werden konnten.

Die Gruppe der 45- bis 60-Jährigen war mit 22 Probanden zu klein, um gesicherte Daten zu erhalten. Die bisher untersuchte Stichprobe zeigt aber eine deutliche Tendenz. Derzeitige Daten lassen davon ausgehen, dass mit 8 der 13 Items die Norm erfüllt würde. Die 2 Probanden in der Altersgruppe von 60 bis 74 Jahre schafften jeweils 6 Items. Aussagen über die Wertigkeit verbieten sich zu diesem Zeitpunkt.

## Diskussion

Die vorliegende Untersuchung wurde durchgeführt, um eine Versorgungsoptimierung für Patienten mit Unsicherheiten der aufrechten Körperhaltung zu ermöglichen. Es gibt eine Vielzahl von Assessmentsystemen, die Gang- und Standunsicherheiten detektieren können. Nicht alle sind evaluiert. Einige sind nonapparativ, weitere mit hohem technischem Aufwand behaftet und andere wiederum als Fragebogensysteme ausgelegt. Im Vergleich mit dem JESS-Score seien hier 5 davon beispielhaft genannt:

### Balance Error Scoring System (BESS)

Dieser Score ist einfach anzuwenden, bedarf wenig materiellen Aufwands und ist zeitlich überschaubar. Durch Standübungen auf weichen Matten in verschiedenen Ausgangspositionen können Aussagen über die posturale Stabilität getroffen werden. Individuelle Informationen zu den möglichen Ursachen pathologischer Testergebnisse sind nicht möglich. Die klinische Anwendbarkeit ist gut, der Zeitaufwand gering. Ob dies auch für die Anwendung bei älteren Probanden gilt, bleibt offen und sollte infrage gestellt werden [3].

### Time-up-and-go-Test (TuG)

Es handelt sich um einen einfachen und schnell auszuführenden Test, der als Ergebnis die Möglichkeit bietet, die allgemeine Mobilität sowie das individuelle Sturzrisiko v. a. Älterer abzuschätzen. Therapeutische Konsequenzen hinsichtlich der Behandlungsstrategie zur Sturzprävention bieten sich bei diesem Setting nicht. Mit dem Stereotyp des Stehens hat dieser Test nichts zu tun [43].

### Y-Balance-Test oder auch modifizierter Star-Excursion-Test (mSEBT)

Dieser Test wurde zur Detektion von Kraft‑, Koordinations- und Beweglichkeitsdefiziten sowie muskulären Dysbalancen entwickelt. Der Testaufbau ist einfach, kann sogar selbst nachgebaut werden und ist nach Adaptation der initial beschriebenen Ausführung auch schnell durchzuführen. Eine Grundsportlichkeit ist notwendig. Für ältere Personen mit Gleichgewichtsdefiziten ist der Test ungeeignet. Ein guter Testleiter kann bei diesem Test möglicherweise Rückschlüsse zur gezielten Beübung des Patienten ziehen. Konkret ablesbar ist dies aber nicht [2, 16, 51].

### Berg Balance Scale (BBS)

Dieser Test soll Aussagen zum Sturzrisiko ermöglichen. Die benötigte Zeitdauer wird mit ca. 20 min angegeben. Diverse Items werden abgeprüft. Der Einfluss der visuellen und propriozeptiven Afferenzen auf das Gleichgewicht kann abgeschätzt werden [4, 47].

### Balance Evaluation Systems Test (BESTest)

Für die Entwicklung dieses Tests waren ähnliche Beweggründe wie für die Erarbeitung des JESS-Scores maßgebend [20]. Mit seinen 36 Items evaluiert diese Testanordnung ebenfalls verschiedene Fähigkeiten mit dem klinischen Effekt der Balance bzw. posturalen Kontrolle. Die Testung erfolgt aufwendig und liegt laut Autoren im zeitlichen Rahmen von 30 min. Die Anforderungen in der Testanordnung sind aber so hoch, dass bezweifelt werden darf, ob diese 30-min-Testzeit tatsächlich ausreichend sind. In ihrem Kräfte- und Allgemeinzustand reduzierte Patienten scheinen häufig mit den Anforderungen überfordert. Räumliche und apparative Voraussetzungen müssen aber gegeben sein. Beispielsweise sind eine Rampe, ein Stepper und freie Gewichte notwendig. Der BESTest nutzt evaluierte Elemente wie den Time-up-and-go-Test, Einbeinstandtest und Gehversuche. Die Grundidee ist als sehr durchdacht einzustufen, die Umsetzung für erwachsene Patienten verschiedener Altersklassen zumindest zu hinterfragen.

Der JESS-Score orientiert sich an verschiedenen Grundfertigkeiten der Bewegung, sog. motorischen Hauptbeanspruchungsformen. Wird der Stand nicht als passiver Zustand, sondern als aktiver Prozess interpretiert, sind alle Fertigkeiten wichtig [8]. Die Prüfung in einzelnen Fähigkeiten erscheint hier nur in Kombination sinnvoll, denn das klinische Ergebnis ist der Stand und keine spezielle Begabung oder Aufgabe. Eine strikte Trennung der Fertigkeiten ist nicht möglich. Hohmann zeigt die Mischung koordinativer und konditioneller Fähigkeiten auf [19]. In Kombination etablierter klinischer Untersuchungen mit selbst entwickelten Testverfahren soll somit ein einfach anzuwendendes Tool geschaffen werden, das dem Untersucher die Möglichkeit gibt, bei nachgewiesenen Defiziten des stabilen Standes gezielt therapieren zu können. Nach Erarbeitung des Scores als theoretisches Grundgerüst wurde für alle neu entwickelten Testverfahren eine Evaluation durchgeführt. Anschließend wurden die Normwerte für den Gesamtscore generiert.

Die gleichwertige Aufstellung der Einzelitems resultiert aus den verschiedenen motorischen Fertigkeiten, die für eine dynamische Stabilisierung des Bewegungssystems notwendig sind. Damit sind v. a. die myofaszialen Strukturen des Körperstammes sowie der unteren Extremitäten gemeint, die den aufrechten Stand und Gang ermöglichen [40, 41].

Neben den bereits beschriebenen grundlegenden koordinativen Fähigkeiten [35] differenziert sich die Propriozeption weiterhin in einen Stellungs‑, Bewegungs- und Kraftsinn [26]. Ohne die Kombination oder bei partiellen Defiziten auch Kompensation aller motorischen Qualitäten kann das Stehen als motorischer Stereotyp nicht physiologisch gelingen. Strukturelle Überbelastung und/oder Schmerzen sind dann die Folge.

**Figure 4:**
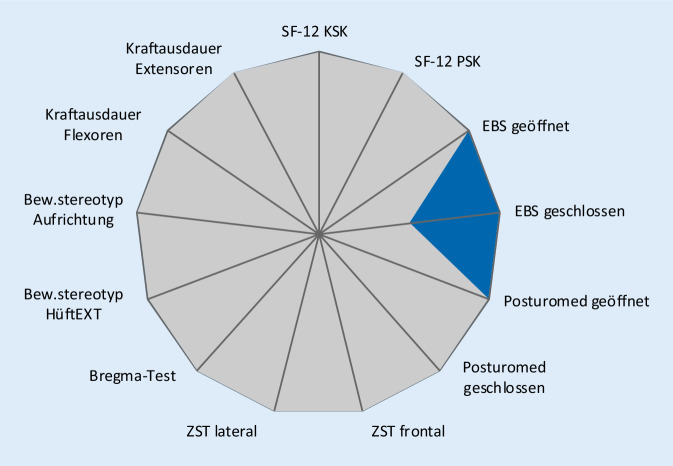

Für die Entwicklung des JESS-Scores war es wichtig, dass neben der einfachen Durchführung ebenfalls eine unkomplizierte Auswertungsoption geschaffen wurde. Cluster und Zahlen erschienen bei 13 Einzelitems nicht zielführend. Alternativ kamen Säulen- und Netzdiagramme infrage. Wegen der besseren Übersicht fiel die Entscheidung auf ein Netzdiagramm. Dabei werden die Normwerte als untere rote Farbfläche und die Testwerte des Probanden grau darüber dargestellt. Die verschiedenfarbige Kolorierung der Norm- und Testwerte erlaubt das sofortige Erkennen von individuellen Defiziten, da diese Werte mittels Signalfarbe bestehen bleiben [33, 37, 52]. Die Abb. 4 und 5 zeigen 2 Beispiele für den Auswertungsreport.

**Figure 5:**
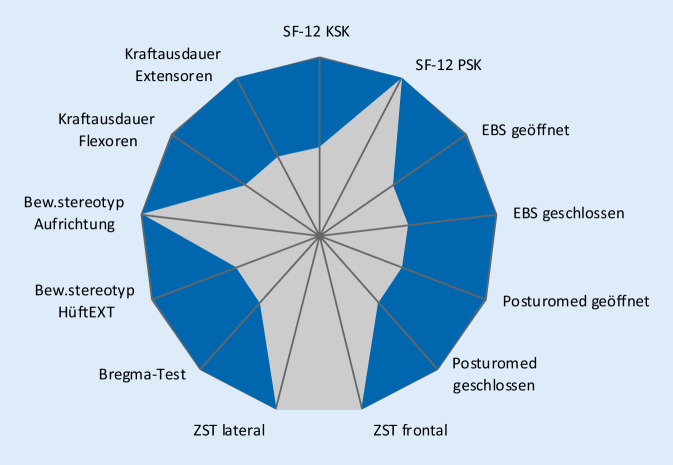

Der motorische Untersuchungsgang beginnt mit dem EBS und der Provokationsposturographie, gefolgt von den oben angegebenen Testeinheiten. Am Ende absolviert der Proband die Kraftausdauertests. Bei diesem Testaufbau kann ein evtl. Einfluss durch Ermüdung vermieden werden. Aktuell ist noch nicht geklärt, welche Rolle die zentrale Ermüdung vor der peripheren spielt. Jedoch kann durch eigene Beobachtungen im Rahmen ärztlicher Diagnostik und Therapie die Entscheidung zu dieser Testreihenfolge vertreten werden. Außerdem besagen klassische Trainingsprinzipien, dass koordinative Einheiten vor Ausdauer- bzw. Krafteinheiten einzuplanen sind. Diese Vorgaben dienten zur Orientierung [15]. Beispielhaft zeigt eine Aufwärmanleitung des Fußballweltverbandes FIFA, wie die klassische Umsetzung erfolgen kann [44, 54, 59].

Die untersuchten Probanden zeigten sich während des Tests durchgehend motiviert. Die primär ausgeführten sensomotorischen Testanteile wurden eher als spielerische Geschicklichkeitstests wahrgenommen. Es hat sich bewährt, die Tests nach Ito und McGill als Letzte auszuführen. Nach Absolvieren dieser anstrengenden Testanteile schienen die Probanden weitgehend erschöpft. Es bleibt zu vermuten, dass eine andere Testabfolge v. a. für die sensomotorisch anspruchsvollen Einheiten negative Auswirkungen haben könnte.

Bei der Entwicklung des JESS-Scores war es wichtig, einen einfachen Test zu erarbeiten. Durch die Berücksichtigung der mit dem Alter zunehmenden sensomotorischen Defizite und in Anlehnung an die Arbeiten von Meinel und Schnabel [35] gelang es, einen Score zu entwickeln, der nicht unter einer direkter Altersabhängigkeit und somit mehreren Auswertungsroutinen leidet. Bei gleichen Anforderungen und Altersunabhängigkeit, differiert lediglich die Anzahl erfolgreich durchgeführter Testitems. Es gilt als gesichert, dass jenseits des 60. Lebensjahres sensomotorische Fähigkeiten durch Alltagsbelastungen erhalten bleiben können und damit durchaus eine Kompensation anderer, verloren gegangener Fähigkeiten auftritt [21]. Ebenso besteht eine große Spanne motorischer Leistungsstufen im Alter [28].

Damit ist es zulässig, die Kraftausdauertests des JESS-Scores auch für höhere Altersstufen im Test zu belassen, wissend, dass diese schwer zu schaffen sind. Durch Kompensation sensomotorischer Fertigkeiten wie dem ZST und dem EBS-Test können diese Defizite ausgeglichen werden. Dies sollte sich dann auch im Score niederschlagen. Selbstverständlich wird es ältere Menschen geben, die wesentlich mehr Anforderungen erreichen, als derzeit im JESS-Score für ihre Alterskategorie genannt sind. Ursächlich ist die sensomotorisch sehr heterogene Gruppe der älteren Menschen. Die Leistungsunterschiede sind bedingt durch diverse Lebensgewohnheiten und Aktivitätsmuster [25]. Es steht fest, dass Alltagsaktivitäten der Abnahme von Organfunktion und körperlicher Leistungsfähigkeit förderlich sind und dieser entgegenwirken [36]. Demgegenüber zeigt sich, dass Krafttraining keinen positiven Effekt auf die Minderung des Sturzrisikos hat [32]. Es kann aber gelingen, durch allgemeine Bewegung im Alltagsleben die „[…] körperliche bzw. psychische Leistungsfähigkeit zu bewahren und von fremder Hilfe unabhängig zu leben“ [1]. Entscheidend ist, Werte für die Durchschnittsbevölkerung zu finden, die nicht immobilisiert ist, aber auch nicht leistungsorientiert Sport betreibt. Es gilt als gesichert, dass sich der alte Mensch in seinem Anspruchsdenken den abnehmenden Fertigkeiten anpasst [18] und viele über 75-Jährige kaum noch sportlicher Betätigung nachgehen [57]. Die große Gruppe der Menschen, die im Alter deutliche Defizite haben, wird deswegen dominieren. Es bleibt zu vermuten, dass bei den derzeit eruierten allgemein koordinativen Fähigkeiten der Schulkinder die Gruppe an sturzgefährdeten, sensomotorisch nur eingeschränkt befähigten Menschen in einigen Jahrzehnten heranwächst [27]. Auch deswegen ist es notwendig, nach Probanden zu suchen, die einen aktiven Lebensstil haben, ohne zur Gruppe der Sportler zu gehören, und mit diesen die Normwerte für höhere Altersstufen zu erarbeiten.

Der JESS-Score kann schon jetzt auch die motorisch deutlich reduzierten Fähigkeiten der Untersuchten abbilden. Besonders geschickte Menschen finden bei den an sie gestellten Anforderungen dennoch Herausforderungen. Bisher unveröffentlichte Daten zeigen, dass Sportarten wie beispielsweise Beachvolleyball überdurchschnittlich häufig dazu befähigen, alle Items des JESS-Scores erfolgreich zu absolvieren. Eine besonders herausragende Position nehmen hier semiprofessionelle Tänzer ein. Diese Gruppe schafft gewissermaßen immer alle Anforderungen.

Der Score muss sich als Instrument zur Verlaufskontrolle durch gezielte therapeutische Intervention erst noch beweisen. Ebenfalls sind die Kohorten in den Altersabschnitten jenseits des 44. Lebensjahres noch nicht umfangreich genug. Gemessen wurden bislang Probanden, die weder Einschränkungen neurologischer oder orthopädischer Art hatten, damit diese sich nicht negativ auf die Testergebnisse auswirken könnten. Zur Vermeidung der Verfälschung der Testergebnisse wurden auch Menschen, die mehr als 2 h Sport pro Woche ausüben, ausgeschlossen. Alltagsaktivitäten wurden hierbei nicht berücksichtigt. Der Weg zur Arbeit mit dem Rad oder zu Fuß fällt nicht unter die Ausschlusskriterien. Im Seniorenbereich Probanden zu finden, die diesen Anforderungen gerecht werden, stellt sich als äußerst schwierig heraus. Es verbietet sich, ältere Studienteilnehmer einzuschließen, die einen Gelenkersatz, bekannte polyneuropathische Beschwerden oder schwerwiegende operative Eingriffe in der Anamnese aufweisen. Probanden, die regelmäßig Sport treiben, können ebenfalls nicht eingeschlossen werden, da diese wahrscheinlich deutlich bessere Ergebnisse zeigen werden, als die der „normale“ ältere Mensch üblicherweise haben würde. Fraglich ist ein evtl. Ausgleich der Extreme, würden beide Gruppen eingeschlossen werden. Darüber kann hier nur gemutmaßt werden. Es ist davon auszugehen, dass die Rekrutierung der Altersgruppe von 45 bis 59 Jahren mittelfristig abgeschlossen werden kann. Die Einbeziehung der Gruppe der über 60-Jährigen wird noch längere Zeit in Anspruch nehmen. Die strikte Einhaltung der Einschlusskriterien ist zwingend zu fordern, selbst wenn es Jahre dauern sollte, geeignete Probanden zu einer statistisch belastbaren Gruppengröße zusammenzufassen, um Verzerrungen durch zu weiche Einschlusskriterien zu vermeiden. Die untersuchten Probanden zeigten häufiger Kraftausdauerdefizite bei den Muskeln der vorderen myofaszialen Kette als bei den Muskeln der eher in Extension arbeitenden. Frey et al. konnten an Pflegekräften zeigen, dass die Rate der Rückenschmerzpatienten von der Ausdauerleistungsfähigkeit der Rückenmuskulatur abhängig ist [14]. Die hier untersuchten Probanden waren gesund. Eine nahezu physiologische Rückenstreckmuskulatur ist dafür wichtig. Frey et al. konnten auch zeigen, dass das zunehmende Alter ein Risiko für das Ausbilden eines Rückenschmerzsyndroms darstellt. Die hier Untersuchten waren zu einem Großteil jünger als 45 Jahre. Möglicherweise sind auch dadurch Einflüsse geltend zu machen. Am ehesten sind die Differenzen zwischen Beuge- und Streckmuskulatur ausschlaggebend und nicht die Absolutwerte [45], wenngleich die Balance der Agonisten/Antagonisten natürlich zu einer gegenseitigen Beeinflussung führt [23, 46]. Ab welchen Dysbalancewerten klinisch relevante Auswirkungen zu erwarten sind, bleibt weiterhin unklar. Dies könnte auch erklären, warum trotz nachgewiesener Defizite in beiden Ketten bei 14 Probanden sich diese dennoch als gesund beschrieben. Die Kraftausdauerfähigkeit ist reduziert. Die Balance der beteiligten Muskelgruppen aber möglicherweise nicht gravierend ausgeprägt. Im individuellen Anforderungsprofil der Untersuchten könnte dies kompensiert sein und somit klinisch nicht auffallen.

## Schlussfolgerung

Die Anwendung des JESS-Scores hat sich bewährt. Der Zeitaufwand ist vertretbar. Die Summe der Tests greift einen großen Bereich sensomotorischen Könnens ab. Es ist möglich, Menschen mit geringen und mit ausgeprägten koordinativen und sensomotorischen Fähigkeiten zu testen und adäquat zu beurteilen. Es braucht in den Altersgruppen jenseits des 44. Lebensjahres größere Stichproben. Der nächste Schritt resultiert dann in einer Kurzform des Scores. Somit soll die Nutzung des Screenings für den Stereotyp des Stehens großflächig ermöglicht werden.
